# Improved biological methanation using tubular foam-bed reactor

**DOI:** 10.1186/s13068-024-02509-1

**Published:** 2024-05-15

**Authors:** Hoda Khesali Aghtaei, Robert Heyer, Udo Reichl, Dirk Benndorf

**Affiliations:** 1 Max Planck Institute for Dynamics of Complex Technical Systems, Bioprocess Engineering, Sandtorstraße 1, 39106 Magdeburg, Germany; 2 Bioprocess Engineering, Otto von Guericke University Magdeburg, Universitätsplatz 2, 39106 Magdeburg, Germany; 3 Database and Software Engineering Group, Otto von Guericke University, Universitätsplatz 2, 39106 Magdeburg, Germany; 4 Faculty of Technology (TechFak) Bielefeld University, Universitätsstraße 27, 33615 Bielefeld, Germany; 5 Multidimensional Omics Analyses group, Leibniz-Institut für Analytische Wissenschaften–ISAS–e.V., Bunsen-Kirchhoff-Straße 11, 44139 Dortmund, Germany; 6 Applied Biosciences and Process Engineering, Anhalt University of Applied Sciences, Bernburger Straße 55, 1458, 06366 Köthen, Germany

**Keywords:** Biomethane, Biological methanation, Hydrogen starvation, Polymeric non-ionic surfactant, Power storage, Power to gas, Tubular foam-bed reactor, Variable renewable energy

## Abstract

**Background:**

Power-to-gas is the pivotal link between electricity and gas infrastructure, enabling the broader integration of renewable energy. Yet, enhancements are necessary for its full potential. In the biomethanation process, transferring H_2_ into the liquid phase is a rate-limiting step. To address this, we developed a novel tubular foam-bed reactor (TFBR) and investigated its performance at laboratory scale.

**Results:**

A non-ionic polymeric surfactant (Pluronic^®^ F-68) at 1.5% w/v was added to the TFBR’s culture medium to generate a stabilized liquid foam structure. This increased both the gas–liquid surface area and the bubble retention time. Within the tubing, cells predominantly traveled evenly suspended in the liquid phase or were entrapped in the thin liquid film of bubbles flowing inside the tube. Phase (I) of the experiment focused primarily on mesophilic (40 °C) operation of the tubular reactor, followed by phase (II), when Pluronic^®^ F-68 was added. In phase (II), the TFBR exhibited 6.5-fold increase in biomethane production rate (*MPR*) to 15.1 $({\text{L}}_{{\text{CH}}_{4}}\text{/}{\text{L}}_{\text{R}}\text{/d)}$, with a CH_4_ concentration exceeding 90% (grid quality), suggesting improved H_2_ transfer. Transitioning to phase (III) with continuous operation at 55 °C, the *MPR* reached 29.7 ${\text{L}}_{{\text{CH}}_{4}}\text{/}{\text{L}}_{\text{R}}\text{/d}$ while maintaining the grid quality CH_4_. Despite, reduced gas–liquid solubility and gas–liquid mass transfer at higher temperatures, the twofold increase in *MPR* compared to phase (II) might be attributed to other factors, i.e., higher metabolic activity of the methanogenic archaea.

To assess process robustness for phase (II) conditions, a partial H_2_ feeding regime (12 h 100% and 12 h 10% of the nominal feeding rate) was implemented. Results demonstrated a resilient *MPR* of approximately 14.8 ${\text{L}}_{{\text{CH}}_{4}}\text{/}{\text{L}}_{\text{R}}\text{/d}$ even with intermittent, low H_2_ concentration.

**Conclusions:**

Overall, the TFBR’s performance plant sets the course for an accelerated introduction of biomethanation technology for the storage of volatile renewable energy. Robust process performance, even under H_2_ starvation, underscores its reliability. Further steps towards an optimum operation regime and scale-up should be initiated. Additionally, the use of TFBR systems should be considered for biotechnological processes in which gas–liquid mass transfer is a limiting factor for achieving higher reaction rates.

**Supplementary Information:**

The online version contains supplementary material available at 10.1186/s13068-024-02509-1.

## Background

In line with the European Union’s goal of achieving climate neutrality by 2050, a significant increase in the use of renewable energy sources is imperative [1]. Drawing from simulations and optimization across 60 comprehensive studies, it is evident that the electricity storage capacity for 100% renewable energy systems is expected to remain below 6% of the total annual energy demand [2]. The strategic redistribution of electricity across various energy sectors can play a pivotal role in reducing the necessary storage capacity and effectively balancing fluctuations in variable renewable energy sources [2, 3]. An alternative approach, power-to-gas (PtG), offers a means of converting electrical energy into chemical bond energy [3]. The biomethane production process involves the transformation of H_2_ produced from surplus volatile renewable electricity and biogenic or waste CO_2_ into CH_4_ through methanogenesis reactions. Biological methanation (BM) serves as both a storage platform for excess renewable power and an eco-friendly means of utilizing CO_2_ [4]. Furthermore, by enriching biogas containing biogenic CO_2_, it is possible to generate grid-quality CH_4_ ranging from higher than 86 to 97% in different countries, particularly in Germany, where it exceeds 96% [5]. This process facilitated via the activity of hydrogen-consuming hydrogenotrophic methanogens in the BM process. This upgraded biogas, characterized by a high CH_4_ content and low impurities, is known as biomethane and can be readily stored within the gas grid based on countries standards [5], making it a versatile energy source for various sectors [6].

In biological aerobic and anaerobic processes, continuously stirred tank reactors (CSTRs) are standardized. However, when the reactive phases consist of less-soluble gases in aqueous media (e.g., H_2_) the productivity of the process is restricted by the transferability of gases between the liquid and gas phases [7, 8]. The low solubility of H_2_ in the aqueous phase can be compensated by increasing the pressure or concentration gradient during the BM process (> 10 MPa) [9–11]. Over the past decade, numerous gas–liquid contactors have been proposed at the laboratory, bench, and pilot scales to address the limitations of H_2_ gas–liquid transfer, and their advantages and disadvantages in the BM process have been extensively reviewed in the literature [4, 12–14]. Gas holdup and interfacial area are the underlying factors that influence the selection of reactor types [8]. These key parameters can be enhanced by employing intensive mixing (> 700 rpm) within a submerged reactor (CSTR), which effectively reduces the gas bubble size and prolongs presence in the liquid phase [8, 15]. Nevertheless, the industrial viability of this approach is challenged by factors such as the shear stress imposed on methanogenic archaea, reduction in syntrophic interactions within mixed cultures and parasitic energy drain associated with high rotational speeds, all of which warrant scrutiny [16–18].

In contrast to submerged systems, such as CSTRs and bubble columns, where the predominant phase is liquid, fixed-film systems such as packed-tubular reactors and trickle beds have gas as their primary phase [14, 16]. In a system where gaseous substrates predominate, the volume of the solution surrounding the biomass is minimized to provide moisture and nutrients of the biofilm [16]. Therefore, much less energy is required to facilitate the gas–liquid mass transfer.

While tubular reactors (TRs) have not been extensively explored for the BM process, they are well-studied and industrialized for continuous bioprocesses, such as algae production [19, 20]. Extensive discussions in the literature, including their simplicity compared to CSTRs, highlight the advantages of TRs in bioprocesses, offering benefits such as thorough mixing, reduced dead zones and a significantly improved area-to-volume ratio for enhanced mass and heat transfer efficiencies [21–23]. In addition, employing helically shaped tubes and generating secondary radial flow can further enhance mixing intensity [24–26]. The elongated gas travel path facilitates prolonged interactions between syntrophic microorganisms [27], hydrogenotrophic methanogens and gaseous substrates. Another advantage of TR systems is the absence of back-mixing with the feeding substrates. Despite the potential of TRs, to our knowledge, only a few studies have reported the development of TR in the BM process [16, 26]. Savvas et al. studied a lab-scale packed tubular reactor primarily aimed to reduce the liquid phase [16]. The results demonstrated productivity ranging from 20 to 39 ${\text{L}}_{{\text{CH}}_{4}}\text{/}{\text{L}}_{\text{R}}\text{/d} \,$(liter of methane per liter of the reactor per day) and CH_4_ purity ranging from 98 to 50% [16]. These findings are noteworthy when compared to other continuous gas–liquid contactors, especially concerning the production of grid-quality biomethane, which typically requires a purity exceeding 90% [13]. Nevertheless, dead phases, clogging, homogeneity of irrigation and biofilm formation are still problems under investigation in such biofilm systems [8]. Hoffstadt et al. introduced a meandering plug flow reactor configuration with an integrated helical static mixer. In this design, microorganisms travel within the liquid phase, while substrates and the product were conveyed in the gaseous phase, mirroring the principles of a bubble column reactor [26]. This approach substantially mitigated the risk of clogging and offered efficient nutrient support in comparison to biofilm reactors, while also being notably more efficient in terms of energy than CSTRs [26]. This study reported a gas fraction of 29%, which was aligned with simulated data. While the study did not provide direct information on the conversion rates, it did mention achieving a commendable conversion rate to the best of our knowledge [26].

In another context, a liquid foam bed photobioreactor was recently developed to enhance the cost-efficiency of algal production by improving the delivery of CO_2_ and O_2_ to the liquid phase [28]. A comprehensive screening of surfactants considering foaming quality, non-toxicity, algal partitioning and slow biodegradability led to the selection of the non-ionic polymeric Pluronic^®^ F-68 (PU-F68) for the development photobioreactor [29]. Several other studies have also highlighted the promising application of froth/foam bed gas–liquid contactors, where reactive gas–liquid interfaces can limit the process or microbial growth rate [30–32]. The extensive surface area provided by foam-containing systems, relative to other gas–liquid contactors, can facilitate the rate-limiting step of gas–liquid transfer. However, the advantages of surfactant-stabilized foam, such as increased gas holdup and extended gas retention time, may be offset by the accumulation of surfactant at the bubble surface and potentially hindering mass transfer rates owing to the presence of surfactant molecules [33].

The aim of this study is to introduce an enhanced BM system by combining a TR with a stabilized liquid foam bed. The primary objectives were to improve biomethane productivity, maintain biomethane quality for grid injection, and showcase operational flexibility during intermittent cycles. To achieve these goals, a novel laboratory-scale tubular foam-bed reactor (TFBR) was developed by adding PU-F68 to a TR reactor that had been operating continuously at 40 °C for over 3 months. Furthermore, the study evaluated the performance and robustness of the mesophilic TFBR, particularly in response to intermittent H_2_ feeding after approximately 5 months of continuous operation. Subsequently, the mesophilic TFBR was transitioned to thermophilic conditions at 55 °C to investigate the effect of temperature in the presence of a surfactant on the kinetics of the BM process.

## Results and discussion

**Fig. 1:**
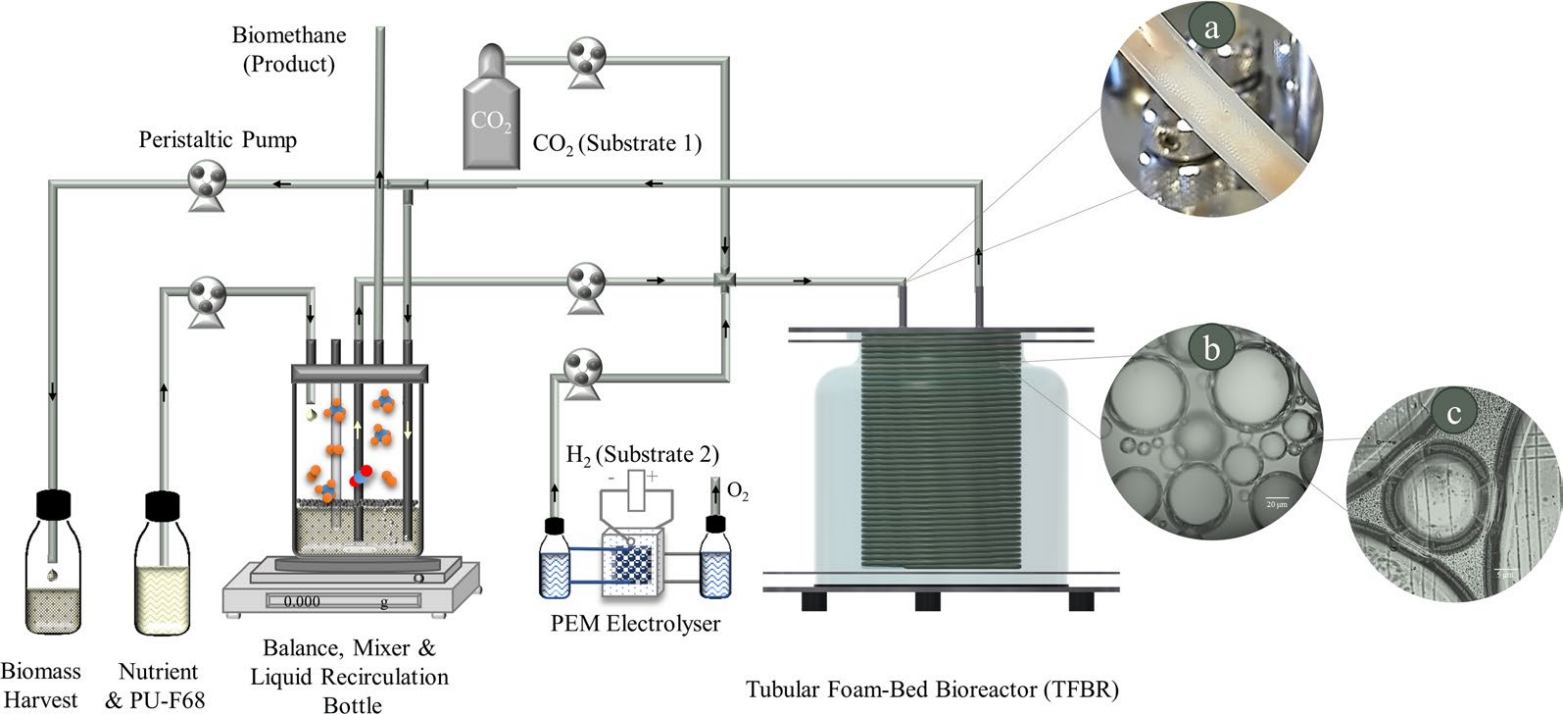
Schematic overview of the tubular foam-bed bioreactor (TFBR) setup for biomethane production. H_2_ was produced via a polymer electrolyte membrane (PEM) electrolyser as in the figure. Stabilized bubbles (foam texture) generated by the addition of Pluronic^®^ F-68 (PU-F68) to the nutrient solution. **a** Foam texture inside the tubing, **b** and **c** light microscopic images of stabilized bubbles taken at 100 × and 400 × magnification, respectively

The BM process spanned a year, using both the TR and TFBR (Fig. 1 and Fig. S.2. Additional file 4). The results from the final stable feeding week of operation are presented below to assess the highest achieved performance of the BM process during these three phases. Furthermore, the robustness of the BM process in such a TFBR system during the partial H_2_ feeding experiment was exclusively investigated in phase (II).

### Biological methanation in the mesophilic tubular reactor: phase (I)

**Fig. 2:**
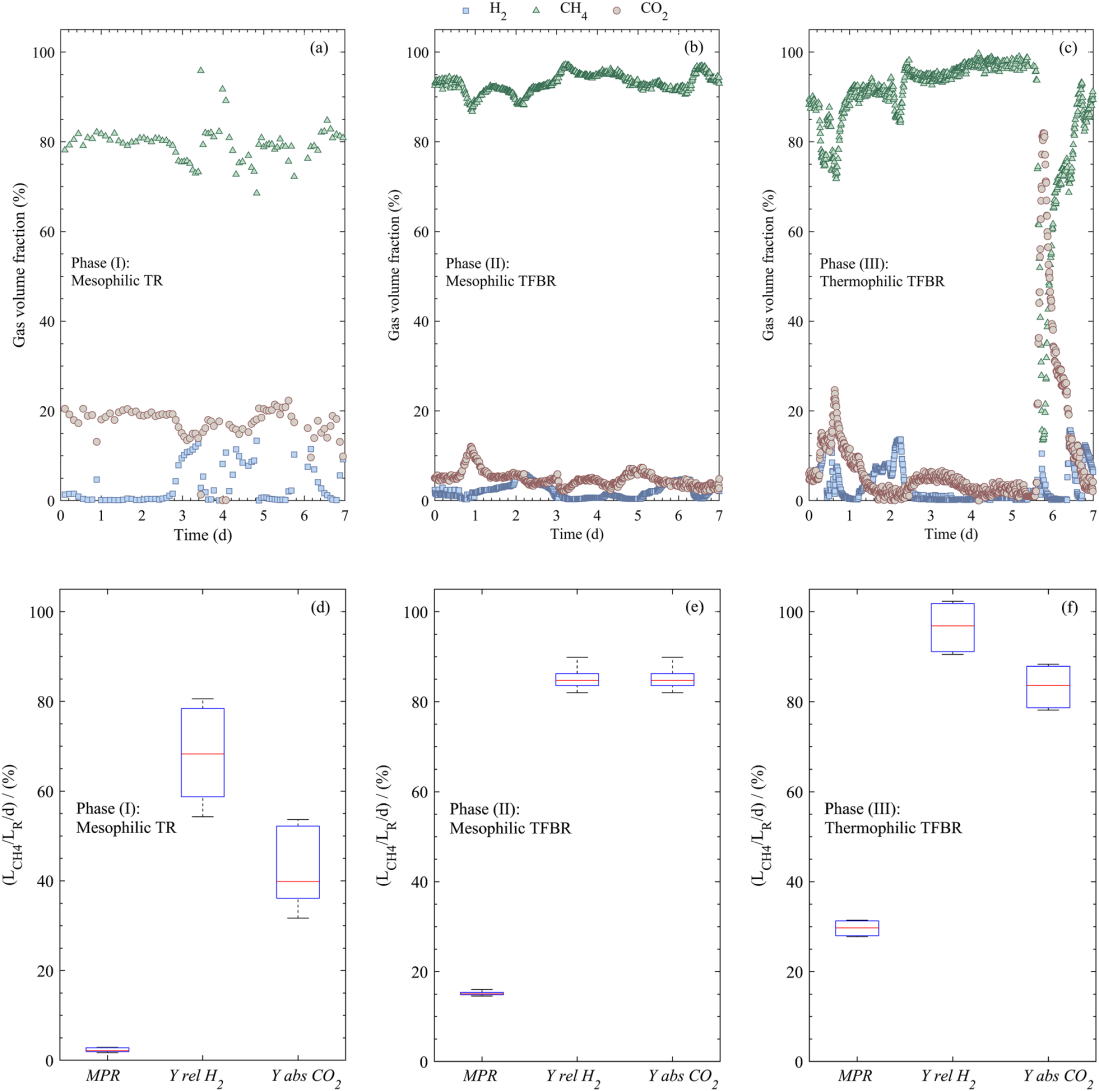
Composition of the gas volume fraction and process performance under different operating regimes. Time course of (**a**) Phase (I): mesophilic tubular bioreactor (TR) at 40 °C; **b** Phase (II): mesophilic tubular foam-bed bioreactor (TFBR) at 40 °C; **c** Phase (III): thermophilic TFBR at 55 °C. Box and Whisker plots for ${\text{Y}}_{\text{rel}}{{\text{H}}}_{2}$ (%), ${\text{Y}}_{\text{abs}}{{\text{CO}}}_{2}$ (%) and *MPR* (${\text{L}}_{{\text{CH}}_{4}}\text{/}{\text{L}}_{\text{R}}\text{/d}$) considering 24 h H_2_ feeding regime for 7 d in **d** Phase (I): Mesophilic TR **e** Phase (II): mesophilic TFBR; **f** Phase (III): Thermophilic TFBR (short-term disturbance at day 5 not considered in calculations)

The experiment was initiated with phase (I) in the mesophilic TR, involving a continuous 3-month biomethanation of CO_2_ and H_2_ (as shown in Table 1). As the biomass grew and adapted to the process conditions, the outlet gas from the TR was consistently monitored and the reactor was fed based on demand (data not shown). The gas profile for the final week of operation indicated an average CH_4_ concentration of 79.4% [based on the total number of samples taken in the presented phase (*n* = 80), standard deviation (SD = 3.9%)], where CO_2_ exhibited variability, averaging 17.0% (*n* = 80, SD = 4.1%) and H_2_ showed an average value of 3.6% (*n* = 80, SD = 4.2%) (Fig. 2a). Excessive CO_2_ feeding and fluctuations resulted from intentional variations in H_2_ feeding, aimed at stabilizing the maximum CH_4_ production while maintaining the H_2_ concentration below the detection limit of the gas analysis system. As a higher amount of H_2_ feeding resulted in the presence of unconverted H_2_ in the headspace, a stoichiometric feeding ratio of CO_2_ and H_2_ could not be achieved using TR. The methane production rate (*MPR)* for the final week was 2.3 ${\text{L}}_{{\text{CH}}_{4}}\text{/}{\text{L}}_{\text{R}}\text{/d}$ (*n* = 7, SD = 0.5 ${\text{L}}_{{\text{CH}}_{4}}\text{/}{\text{L}}_{\text{R}}\text{/d}$), with ${\text{Y}}_{\text{rel}}{{\text{H}}}_{2}$ of about 68.4% (*n* = 7, SD = 10.0%) and ${\text{Y}}_{\text{abs}}{{\text{CO}}}_{2}$ of 43.4% (*n* = 7, SD = 8.9%) (Fig. 2d). At the end of phase (I), the concentration of suspended biomass dry weight reached approximately 1.5 g/L (The optical density at 600 nm, OD_600_ = 2.7) [16]. The findings of this study in phase (I) within the TR were comparable in terms of the *MPR* to other studies using fixed or submerged reactors [6, 13]. However, they still fall short of the reported high-performance continuous systems, such as trickle beds (15.4 ${\text{L}}_{{\text{CH}}_{4}}\text{/}{\text{L}}_{\text{R}}\text{/d)}$, packed tubular (27 ${\text{L}}_{{\text{CH}}_{4}}\text{/}{\text{L}}_{\text{R}}\text{/d}$) and cascade reactors (36 ${\text{L}}_{{\text{CH}}_{4}}\text{/}{\text{L}}_{\text{R}}\text{/d}$) [16, 34, 35].

**Table 1:** Operation regimes for different process phases. Pluronic® F-68 (PU-F68) was added as a surfactant to produce a liquid foam at w/v (%)

Period	Reactor name (abbreviation)	H_2_ feeding regime	Time (d)	T (°C)	PU-F68 (%)
Phase (I)	Tubular reactor (TR)	24 h (100%)^†^	92	40	0
Phase (II)	Tubular foam-bed reactor (TFBR)	24 h (100%) 12 h (10%)–12 h (10 %)	174 21	40 40	1.5 1.5
Phase (III)	Tubular foam-bed reactor (TFBR)	24 h (100%)^*^	77	55	1.5

^*^ Short-term disturbance in H_2_ feeding (Fig. 2c) at 5th days not considered

^†^ Percentages in H_2_ feeding refer to the proportional H_2_ feeding based on nominal feeding in percent

**Fig. 3:**
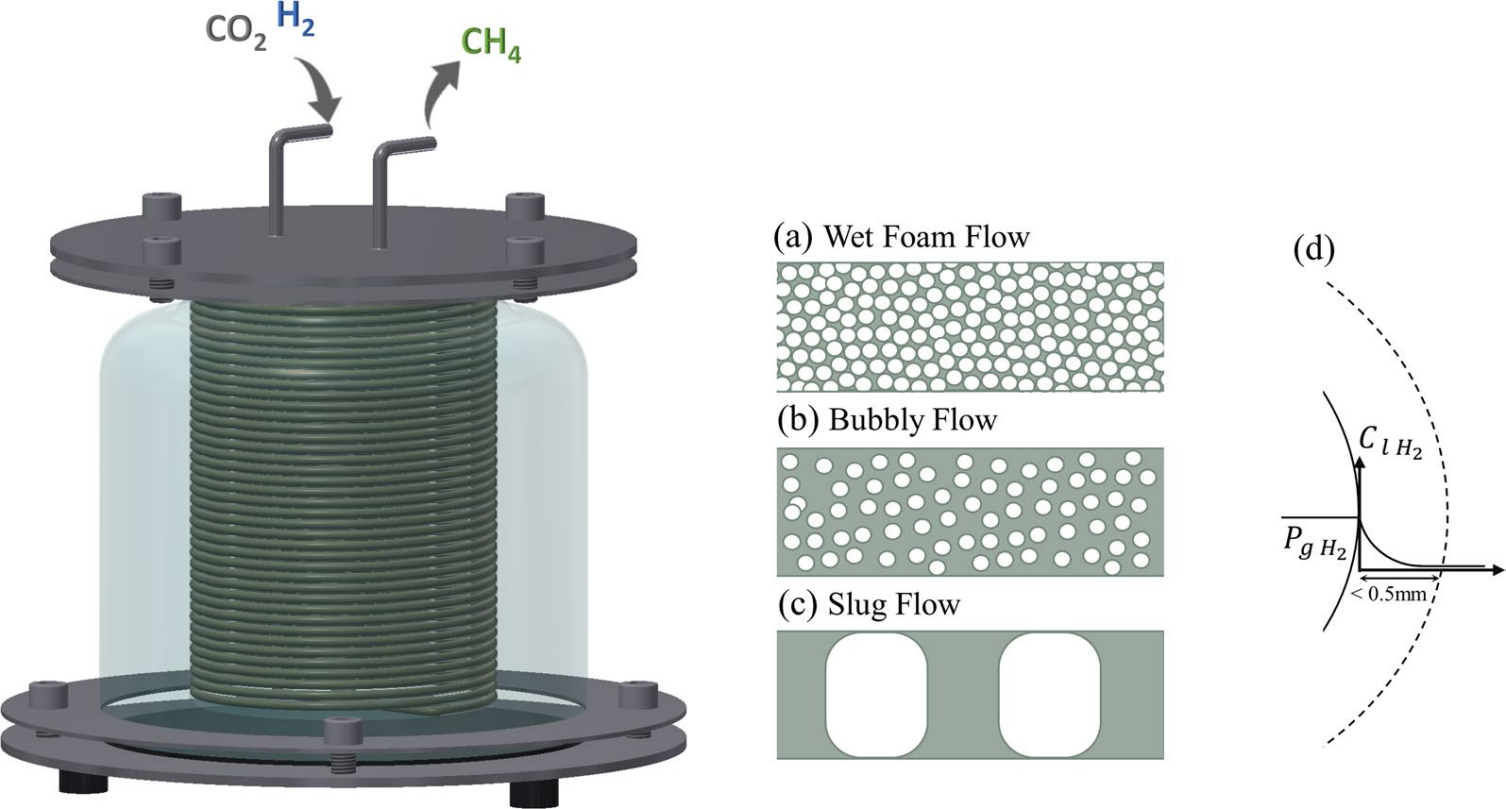
Overview of different flow structures in tubular (foam bed) reactor. The flow structure varies based on the liquid fraction and the presence/absence of the surfactant. **a** Wet foam flow, high-density foam or bubble jamming; **b** bubbly flow; **c** slug flow. **d** H_2_ bubble gradient in an active biological methanation process reported by Garcia-Robledo et al.; Maegaard et al. (part of figure adapted from Jensen et al. [8, 40, 41])

In phase (I) of TR, the gas and liquid flow structures were visually characterized as a segmented two-phase flow along the tube length, specifically exhibiting a slug flow pattern (Fig. 3c) [36]. The presence of stable small bubbles within the flow enhances the gas–liquid interaction, fostering increased mass transfer and system productivity. However, owing to the high surface tension of water, bubbles tend to merge upon collision, leading to gas-filled cavities and a reduction in the gas–liquid interfacial area (bubble coalescence). Moreover, the narrow internal diameter of the tube further increases the likelihood of collision and adhesion tendency of the liquid phase [16]. To address this challenge, stabilizing agents such as surfactants can be introduced, absorbing at the gas–liquid interface and promoting bubble stability [37, 38].

### Biological methanation in the mesophilic tubular foam bed reactor: phase (II)

Complex biological solutions, which include cell debris, proteins, biosurfactants, biopolymers, fatty acids and various essential ingredients for living cells, naturally lead to foam formation when gas is injected through a liquid (i.e., foam formation in bioreactors). Nevertheless, the resilience of the foam and consequently, the efficiency of the gas–liquid mass transfer are intricately affected by numerous factors. These factors include the specific type and concentration of the ingredients, temperature, pressure, flow rheology and degradability of the surfactant [37, 38]. Consequently, PU-F68, a synthetic polymer renowned for its exceptional foaming properties, was strategically selected to establish a stabilized liquid foam within a continuously operating TR [27]. The experiment continued by adding the PU-F68 surfactant to the recirculating liquid phase as a bubble stabilizer and transferring the TR reactor to the TFBR [phase (II) Table 1]. PU-F68 played a pivotal role in reducing the surface tension between the water molecules, thereby enhancing the stability of the micro-bubbles formed after gas injection (Figs. 1a–c and 3a). Beyond the critical micellar concentration (CMC), surfactant molecules aggregate to form micelles, actively contributing to the creation of stabilized bubbles and foam [29]. Following the recommendations of Janoska et al., a concentration of 1.5% (w/v) PU-F68 was deliberately chosen (approximately five times the CMC concentration). This selection aimed to obtain a foam with intermediate stability, roughly translating to a half-life of approximately 1 h [29, 39].

Starting from phase (II), a discernible enhancement in the uniformity of the outlet gas and a notable increase in the *MPR* were observed. The weekly average concentration of CH_4_ was recorded at 93.1% (*n* = 627, SD = 2.1%), with H_2_ and CO_2_ concentrations averaging at 2.1% (*n* = 627, SD = 1.5%) and 4.8% (*n* = 627, SD = 1.6%), respectively (Fig. 2b). The average weekly *MPR* experienced an impressive 6.5-fold increase compared to phase (I), reaching a value of 15.1 ${\text{L}}_{{\text{CH}}_{4}}\text{/}{\text{L}}_{\text{R}}\text{/d} \,$(*n* = 7, SD = 0.4 ${\text{L}}_{{\text{CH}}_{4}}\text{/}{\text{L}}_{\text{R}}\text{/d}$) (Fig. 2e). Achieving a stoichiometric feeding ratio of 4H_2_: 1CO_2_ (Fig. 2e), the average relative and absolute conversion yields of H_2_ and CO_2_ were calculated at 84.8% (*n* = 7, SD = 2.1%). In contrast to phase (I) in this study, the performance of the mesophilic TFBR was comparable to that of studies conducted in fixed-film/submerged bioreactors under mesophilic conditions reviewed by other studies [13, 40]. To the best of our knowledge, the highest reported laboratory-scale continuous BM’s *MPR* value exceeding 90% CH_4_ concentration was achieved by a cascade packed column reactor (36 ${\text{L}}_{{\text{CH}}_{4}}\text{/}{\text{L}}_{\text{R}}\text{/d}$) running for a limited duration of 37 h due to technical issues [35]. While some studies have reported higher values, they achieved this at the trade-off of methane concentration [6, 13].

The flow structure underwent significant changes following the addition of PU-F68, in contrast to the initial phase (I) within TR and varied along the tube as the BM process progressed. Towards the end of phase (II), where the highly productive BM process took place with a gas-to-liquid feeding ratio of 7:1, the flow can be visually described as follows: at the injection point, the flow predominantly resembled a high-density or wet foam (Figs. 1a, 3a). As the BM process advanced and the gaseous substrates underwent conversion, the bubbles diminished in size and process water was produced. The progression of the BM process along the tube led to an increase in the liquid phase fraction and transition to bubbly flow, where the liquid phase fraction passed the critical bubble jam fraction (Fig. 3b) [37]. Ultimately, at the tube outlet, where the majority of the gaseous substrates were converted, the flow transitioned into slug flow (Fig. 3c). The inherent breakdown of bubbles in the BM process [1CO_2 (g)_ + 4H_2 (g)_ ⟶1CH_4 (g)_ + 2H_2_O _(L)_] prevented a further additional step of foam breakage, which was reported in the literature as a subsequent step aimed at releasing the gas phase trapped within the bubbles [41].

Microscale investigations revealed that the BM process was confined to a narrow zone around the gas–liquid surface (< 0.5 mm) (Fig. 3d) [41, 42]. Consequently, the gas liquid mass transfer limitation was identified as a constraining factor for the BM process in the bulk [42, 43]. Despite the variations in bubble size during the BM process, the utilization of PU-F68 to stabilize the small bubbles along the tube markedly enhanced the BM productivity and conversion yields.

The enhancement in gas–liquid mass transfer resulted in a more efficient process and a higher rate of biomass/nutrient recycling. The biomass density was increased to 9.3 g/L (OD_600_ = 19.6) by the end of the stable running experiment. Although biomass recycling was undertaken under anaerobic conditions to sustain biomass activity, it is acknowledged that this approach may have led to the lysis of certain community members. Existing evidence suggests the advantageous role of biomass lysate recycling in providing essential nutrients during the BM process [27, 35, 44].

### Biological methanation in the thermophilic tubular foam bed reactor: phase (III)

The temperature plays a crucial role in gas–liquid reactions, influencing reaction rates, equilibrium constants, and the overall kinetics of the reaction. The effect of temperature can vary depending on the specific reaction and the reaction conditions. In phase (III), the temperature of the mesophilic TFBR system was increased from 40 to 55 °C. At the outset of experiment, biomass concentration decreased by approximately half as a result of temperature shifts; however, after the adaptation phase, it recovered and reached 10.0 g/L (OD_600_ = 23.5). Towards the end of phase (III), there was a 16-h interruption in the feeding of H_2_, resulting in a significant increase in CO_2_ concentration, while the CH_4_ concentration decreased (Fig. 2c). Upon resuming H_2_ feeding, the TFBR self-recovered within approximately 8 h, demonstrating capability of the reactor to handle H_2_ interruption.

Excluding the explained interruption phase, on average, the outlet gas predominantly consisted of CH_4_ (92.1%, *n* = 588, SD = 6%), with less than 8% H_2_ and CO_2_ (Fig. 2c). The *MPR* in thermophilic TFBR reached the average value of 29.7 ${\text{L}}_{{\text{CH}}_{4}}\text{/}{\text{L}}_{\text{R}}\text{/d}$ (*n *= 7, SD = 1.5 ${\text{L}}_{{\text{CH}}_{4}}\text{/}{\text{L}}_{\text{R}}\text{/d}$ compared to 2.3 and 15.1 ${\text{L}}_{{\text{CH}}_{4}}\text{/}{\text{L}}_{\text{R}}\text{/d}$ in phase (I) and phase (II) respectively (Fig. 2d, e). In addition, H_2_ and CO_2_ conversion yields reached 96.6% (*n* = 7, SD = 5%) and 83.4% (*n* = 7, SD = 4.3%) respectively. Temperature significantly influences the metabolic rate of conversion and the stability of hydrogenotrophic methanogens [6, 45]. Moreover, higher gas–liquid transferability can be achieved at higher k_L_ and lower viscosity, which can be attained at higher temperatures [6]. However, increasing the temperature resulted in a drastic decrease in H_2_ solubility [46]. Temperature also affects various physiochemical properties of the foam, such as surface tension, CMC, bubble coalescence and stability, underscoring its importance in monitoring the BM process performance and stability [38, 39].

Despite the negative effects of temperature on H_2_ solubility and foam stability, the thermophilic-enriched community demonstrated an almost twofold increase in the productivity of the BM process compared to phase (II). Studies on microbial community composition and the effect of temperature have suggested that increasing the temperature to 55 °C promotes the enrichment of hydrogenotrophic methanogens compared to acetoclastic methanogens and other competing bacteria [46, 47]. Profiling the metabolically active community in the samples collected from phases (II) and (III) is necessary for concrete conclusions on hydrogenotrophic methanogens enrichment. Nevertheless, the higher *MPR* observed in phase (III) (i.e., thermophilic TFBR) may be attributed to the greater abundance of hydrogenotrophic methanogens at this temperature. Although thermophilic anaerobic digestion processes have shown higher process performance, temperatures below 55 °C provide an advantage in terms of BM process stability [48, 49]. Higher temperatures can lead to a more fragile process owing to the lower microbial richness and diversity of catabolism [46]. Comparing phases (II) and (III), no visible changes in the flow structure and stability of the foam along the tube were observed. Achieving a higher *MPR* in a TFBR system may involve longer biomass enrichment and optimal system design.

### Demand-oriented biological methanation in phase (II) In the mesophilic tubular foam bed reactor

**Fig. 4:**
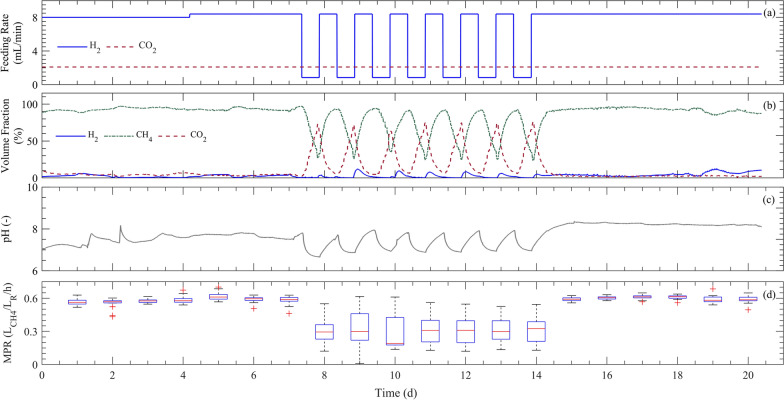
Time course of various process parameters for the mesophilic tubular foam-bed bioreactor using a partial H_2_ feeding regime. **a** Substrate feeding rates; **b** outlet gas composition; **c** pH value; **d** hourly methane production rate (*MPR*)

The flexibility and stability of the BM process are of paramount importance, considering PtG as an alternative storage technology for surplus renewable electricity [27, 50]. In the scheduled 3-week experiment during phase (II), the mesophilic TFBR was subjected to repeated partial H_2_ feeding, as shown in Fig. 4a. Previous research has highlighted the advantages of supplying minimal H_2_ feeding compared to the nominal value, preventing variations in microbial community structure when resources are limited; therefore, a shorter recovery time is needed [27, 50]. A recent microbial study revealed a distinct resource allocation strategy utilized by a methanogenic archaeon under conditions of energy scarcity, diverging fundamentally from the well-studied versatile chemoheterotrophic bacteria like E. coli [51]. This study has shown that methanogens maintain their proteome allocation even at low H_2_ fluxes, suggesting their capability for rapid recovery [51]. This strategy was also applied in the present study, to avoid either structural or metabolic activity loss during downtime (starvation period).

In the first week of stable operation, the outlet gas maintained a consistent CH_4_ content of approximately 93% on weekly average (Fig. 4b). However, upon initiating partial H_2_ feeding, the amount of non-converted CO_2_ increased, resulting in a decrease in CH_4_ content to an average of 30% within 7 days of the middle week (Fig. 4b). The pH slightly fluctuated with H_2_ feeding changes (average pH = 7.65 ± 0.45), representing the buffering capacity of the liquid phase in capturing the unconverted CO_2_ during downtime (Fig. 4c). Upon resuming H_2_ feeding, the conversion of CO_2_ to CH_4_ was promptly recommenced and the CH_4_ content reached its highest value within the 12 h (Fig. 4b). In the third week, with continuous nominal feeding of H_2_, no process disturbances were detected, indicating resilience against H_2_ fluctuations experienced in the previous week.

A comparison between the first and second weeks, as depicted in Fig. 4d, reveals that H_2_ fluctuations contributed to a decline in the hourly *MPR* and its instability (to demonstrate the variation caused by partial H_2_ feeding, the *MPR* is reported here on an hourly basis intentionally). As H_2_ feeding was reduced by almost half during the second week, the daily *MPR* also halved; however, in the third week, it regained stability and reached the same level as in the first week. While the H_2_ conversion yield remained relatively stable, the absolute CO_2_ conversion yield was reduced by half due to H_2_ deficiency (Additional file 3, Fig. S.1). Furthermore, the third week demonstrated even more stability in terms of the process results. According to a previous study on the microbial community responses to H_2_ fluctuations in the BM process, an increase in the abundance of specific methanogens could be possible [27]. Therefore, the BM process in the mesophilic TFBR exhibited robustness against partial H_2_ feeding and its implementation was expected to be demand-oriented. However, to draw conclusions on the suitability of this system for coupling with the renewable energy sector, more evidence regarding non-linear changes in H_2_ feeding, aligned with the availability profile and price of the surplus electricity, is needed [50]. Thus far, the BM process systems have demonstrated a unique ability for demand-oriented operation in mild conditions, characterized by controllability and rapid response to changes, distinguishing them from physiochemical processes [13, 52]. Studies on flexibility have indicated that hydrogenotrophic methanogens are generally considered as being resilient and robust against dynamic operation of the BM process [50]. However, literatures have also reported microbial structure or activity changes and their consequential effects on performance during downtime periods [27, 34, 53, 54]. Recovery-time to reach the peak of performance is dependent on several factors, including downtime temperature, duration and feeding gas flow [50]. Optimizing these factors plays a crucial role in the demand-oriented operation of various BM reactor systems.

### Perspective and challenges of tubular foam-bed reactor

While previous studies have primarily focussed on preventing foam production in their BM systems [15, 55], the present study uniquely aimed to stabilize micro-bubbles and foam production to enhance the transferability of H_2_ to the liquid phase. In comparison to the other studies, the laboratory-scale TFBR demonstrated promising and comparable results [6, 13]. Burkhardt et al. highlighted significant differences in reactor performance among various concepts, emphasizing the trade-offs between *MPR*, CH_4_ concentration and energy expenditure [13]. They found that while CSTRs can achieve high *MPR* due to their intense energy input for mixing, this comes at the expense of lower final CH_4_ concentration and the need for post-modification steps to reach the grid-quality biomethane [13].

The improvement in gaseous substrates availability, higher conversion rates and biomass recycling facilitated the growth of the microbial community. After adding a polymeric surfactant, the *MPR* in phase (I) improved roughly sevenfold in phase (II) of TFBR, increasing to 13-fold with higher temperature during phase (III). Final gas composition improvements to grid values were also notable in phase (II) and phase (III).

Comparatively, the TFBR achieved a threefold higher *MPR* than an earlier laboratory-study by Electrochaea in 2013 using a CSTR (9.93 ${\text{L}}_{{\text{CH}}_{4}}\text{/}{\text{L}}_{\text{R}}\text{/d}$), with the H_2_ conversion efficiency of about 96% conducted at atmospheric pressure, thermophilic conditions and CO_2_ and H_2_ as substrates [15]. Furthermore, in the same study, they improved the CSTR productivity by increasing the pressure (1.2 atm), resulting in an *MPR* of approximately 47 ${\text{L}}_{{\text{CH}}_{4}}\text{/}{\text{L}}_{\text{R}}\text{/d}$, although with a reduced H_2_ conversion efficiency of about 80% [15].

In the PtG BioCat project pilot studies by the same group in 2017 using a reactor volume of 3.5 m^3^, a pressure of 7.8 atm, a temperature of 62 °C and biogas as the feed (35% CO_2_, 63% CH_4_), they produced approximately 170 ${\text{m}}_{{\text{CH}}_{4}}^{3}\text{/}{\text{m}}_{\text{R}}^{3}\text{/d}$ of grid-based biomethane [4, 56]. Further investigations could explore the feasibility of high-pressure studies in TFBR within a scaled-up setup, prioritizing process safety. Attention should be given to the recycling of media containing PU-F68 at a larger scale to reduce the costs and address its low biodegradability. Considering the technology readiness levels (TRL), our evaluation places this technology in level 3. This stage, involves testing the TFBR prototype in a laboratory and controlled environment to confirm that it operated as intended. While level 3 signifies an achievement in proving the concept’s feasibility, additional development and testing are typically necessary to advance the technology to higher TRLs.

Exploring the challenges and opportunities of the TFBR, it becomes paramount to delve into the implications of incorporating a surfactant. The investigation should not only encompass the economic and environmental aspects of the surfactant but also should confirm the observed resilience in anaerobic digestion, in general. Furthermore, the dilution of surfactant due to metabolic water production associated with biomethanation should be considered.

Therefore, dedicated studies are warranted to meticulously examine the potential of recycling the liquid phase containing nutrients and PU-F68. Maybe, ultrafiltration could apply to remove water without loosing PU-F68. Concurrently, a vigilant assessment of the long-term impact of the polymeric surfactant on the microbial community is imperative, ensuring the robustness and stability of the biological methanation process. Furthermore, directing additional research efforts towards optimizing gas injection methods to the TFBR can effectively address the pressure drop issue, particularly in large-scale applications. These synergistic endeavors collectively will propel the TFBR’s TRL and underscore its potential as a key player in advancing biomethanation technology within the renewable energy landscape.

## Conclusions

In a summary, a distinctive approach was implemented to address the issue of H_2_ mass transfer to the liquid phase of the BM process by utilizing stabilized liquid foam. This innovative strategy significantly improved the productivity of the tubular system, achieving an output of approximately 30 ${\text{L}}_{{\text{CH}}_{4}}\text{/}{\text{L}}_{\text{R}}\text{/d}$ under thermophilic conditions, while maintaining its grid-base quality. The promising outcome of the partial H_2_ feeding experiment underscored the flexibility and capacity of the TFBR for dynamic energy conversion. Further studies to support the scale-up of the TFBR are crucial for advancing its potential applications.

## Materials and methods

### Chemicals and nutrient solutions

The composition of the nutrient solution for microorganism growth is listed in supplementary material, Table S.1. To generate stable liquid foam in the TFBR, a non-ionic polymeric surfactant, Pluronic® F-68 BioChemica (PanReac Applichem, Darmstadt, Germany), was used at a concentration of 1.5% w/v.

### Reactor setup and operation conditions

As shown schematically in Fig. 1, the setup primarily consisted of a TR, a liquid recirculation bottle and a polymer electrolyte membrane (PEM) water electrolyzer. CO_2_, one of the main substrates for the BM process, was supplied from a purchased gas bottle (CO_2_ 4.8, Linde Gas GmbH, Pullach, Germany) while H_2_, the second main substrate of this process, was generated locally, by splitting water molecules to H_2_ and O_2_ using a PEM electrolyzer (E104, Double Electrolyzer, H-TEC Education, College Station, TX, USA).

#### Tubular bioreactor

In this study, the TR (Fig. 1) consisted of a helix-formed nylon pneumatic air tube (13 m long, 4 mm inner diameter, winding core 100 mm, with connections reaching an approximate working volume of 170 mL, VWR, Germany) placed in a 1L jacketed vessel (Eppendorf AG, Hamburg, Germany) and controlled by two BioFlo^®^120 systems (Eppendorf, Inc., Enfield, CT, USA). The vessel’s temperature was maintained at 40 °C under mesophilic conditions and 55 °C under thermophilic conditions using a Pt100 probe. Liquid recirculation, substrate feeding, nutrient feeding and biomass harvest were carried out using a pulse-feeding method with the assistance of BioCommand® (Eppendorf, Inc., Enfield, CT, USA) and two peristaltic pumps from the BioFlo®120 systems. Other unused ports of the main vessel and recirculation bottle were sealed with stainless steel plugs or connected to gastight tubing Santoprene® LEZ–SAN, with an internal diameter of 1.6 mm and a thickness of 1.6 mm (Medorex, Nörten-Hardenberg, Germany), which were closed with a luer/lock sampling valve (Eppendorf AG, Hamburg, Germany). The recirculation bottle in Fig. 1 had a volume of 450 mL and the pH of the reactor was controlled in this bottle using ISM® sensors (Inpro 325X (i), Mettler Toledo^®^, Geissen, Germany).

A total liquid volume of 170 mL, including biomass as BM process biocatalysts, was continuously circulated between the main reactor and the recirculation bottle at a rate of 1.5 mL/min. The liquid level in the recirculation bottle varied based on the feeding rate of gaseous substrates, but stabilized at a fixed level during constant feeding rates. A balance was placed under the liquid recirculation bottle to collect the harvest, which included process water and the supplied nutrient solution.

To achieve high cell density in a short period, the harvest was collected, centrifuged and the biomass was returned to the reactor under anaerobic conditions, as explained in Additional file 1, part S.1. Sampling (12–14 mL) and nutrient feeding were performed through ports on the recirculation bottle.

The gaseous substrates were injected at the tube inlet through an in-line needle injection port. Stabilized bubbles flowed through the tube, as shown in Fig. 1a. The outlet of the tube reached the recirculation bottle, where the processed gas and recirculating liquid were separated. When the headspace of the recirculation bottle reached 80 mbar, the outlet gas was automatically vented, cooled through a gas condenser and further analyzed using a gas analysis system.

#### Reactor operation

The TR setup was operated in three distinct phases, as presented in Table 1. These extended operational periods included phases dedicated to biomass formation and adaptation to improved substrate feeding. In phase (I), the mesophilic TR was inoculated with biomass obtained from an ongoing BM setup conducted in-house (further information is provided in the Additional file 1: part S.1).

Following approximately 3 months of continuous operation, phase (II) commenced with the introduction of PU-F68 (1.5% w/v) into the liquid phase of the mesophilic reactor, resulting in the transformation of the system into a TFBR (Table 1). Phase (II) spanned over a period of more than 5 months.

Towards the end of this phase, the reactor underwent a week-long challenge involving fluctuating H_2_ feeding (referred here as a partial H_2_ feeding experiment). During this experiment, the mesophilic TFBR received nominal H_2_ feeding (approximately 500 mL/h) for 12 h, followed by a subsequent 12 h period where only 10% of the nominal feeding rate (approximately 50 mL/h) was supplied. In phase (III), the reactor temperature transitioned from mesophilic (40 °C) to thermophilic (55 °C) conditions, as explained in the Additional file 1, part S.1.

### Analytics

Gas composition and volume of the outlet gas were continuously monitored using a "GärOnA" system equipped with an integrated gas chromatography (GC) analysis instrument (Gesellschaft zur Förderung von Medizin-, Bio- und Umwelttechnologien e.V. (GMBU), Halle, Germany; mobilGC Elektrochemie Halle GmbH (ECH), Halle, Germany). Further details regarding the specific settings can be found in the reference [27].

The dry weight of biomass was determined using a vacuum filtration unit and Polyethersulfone membrane filters with a pore size of 0.2 μm (Sartorius Stedim Biotech Gmbh, Göttingen, Germany). One mL sample was washed with 13 mL of ultra-pure H_2_O Milli-Q^®^ in a 15 mL Falcon tube, thoroughly mixed and filtered using pre-dried and weighed membrane filters. These filters were then dried at 70 °C in an oven (Kelvitron® t, Heraeus Instruments, Hanau, Germany) until a stable reading was achieved. The OD_600_ was measured using a spectrophotometry method (Thermo Fisher Scientific Inc., Madison, WI, USA) and appropriate dilution was applied to obtain readings between 0.0 and 0.9. The OD_600_ of the undiluted samples was calculated based on the applied dilution factor.

Microscopic images of the foam were captured using a reactor sample and artificial foam generation outside the reactor, through air injection, observed at 100 × and 400 × magnification using a light microscope (Primo Star, Carl Zeiss, Göttingen Germany).

### Process parameter estimations

The process performance was assessed by calculating the *MPR*, relative H_2_ to CH_4_ conversion yield $({\text{Y}}_{\text{rel }}{\text{H}}_{2}$) and absolute conversion yield of CO_2_ to CH_4_ (${\text{Y}}_{\text{abs}}{{\text{CO}}}_{2}$). Detailed explanations of these calculations can be found in Additional file 2, of this study, as explained in the literature [27, 57].

### Supplementary Information

Additional file1: S.1. Inoculation and biomass enrichment supplementary information includes preparation of the inoculum start-up and operation information of the biological methanation tubular reactor during different phases. Table S.1 Composition of the applied 1 × nutrient solution was as follows. To prepare the nutrient solution, a sterilized filtration unit was used. After preparing the nutrient mixture, the solution was either degassed with N_2_ for 5 min or kept overnight in the anaerobic workbench for the media of the tubular foam-bed bioreactor, which included 1.5 % (w/v) Pluronic® F-68 agent. In the final step and before usage, the sterilized Na_2_S·9H_2_O solution was added. In phase (I), the media included 10 % (v/v) formic acid as a co-carbon source.

Additional file 2: S.2. Process parameter estimations include formulas for the calculation of methane production rate and substrate conversion efficiency for biological methanation. It also includes formulas to calculate H_2_ production based on Faraday’s law.

Additional file 3: S.3. Process conversion yield during partial H_2_ feeding experiment includes Fig. S.1 Relative H_2_ conversion yield (${\text{Y}}_{\text{rel}}{{\text{H}}}_{2}$) and absolute CO_2_ (${\text{Y}}_{\text{abs}}{{\text{CO}}}_{2}\text{)} \,$ conversion yield of biological methanation process in response to partial H_2_ feeding experiment in phase (II) within mesophilic tubular foam-bed bioreactor.

Additional file 4: S.4. Process results includes Fig. S.2 Time course of process results over a year considering different phases of the bioreactor. The Box–Whisker plots are produced using the average value of each day for every week. Different phases are separated using dash lines, and partial H_2_ feeding experiments are indicated by dash–dot lines during phase (II). In phase (I), a tubular reactor in mesophilic conditions was operated, whereas a tubular foam-bed reactor was operated in mesophilic and thermophilic conditions in phases II and III, respectively. (a) Daily methane production rate (*MPR*), (b) Volume fraction of CH_4_ in the outlet gas, (c) Loading of CO_2_ (L/d), (d) Loading of H_2_ (L/d).

## Data Availability

The data that support the findings of this study are available from the authors.

## Abbreviations

${\text{Y}}_{\text{abs}}{{\text{CO}}}_{2}$: Absolute conversion yield of carbon dioxide to methane
BM: Biological methanation
${\text{Y}}_{{\text{CH}}_{4}}$: Methane volume fraction in percentage
CMC: Critical micellar concentration
CSTR: Continuously stirred tank reactor
${\text{m}}_{{\text{CH}}_{4}}^{3}\text{/}{\text{m}}_{\text{R}}^{3}\text{/d}$: Cubic meter of methane per cubic meter of reactor per day
${\text{C}}_{{\text{l H}}_{2}}$: Hydrogen concentration in liquid phase
GC: Gas chromatography
${\text{L}}_{{\text{CH}}_{4}}\text{/}{\text{L}}_{\text{R}}\text{/d}$: Liter of methane per liter of reactor per day
k_L_: Mass transfer coefficient in liquid
*MPR*: Methane production rate
n: Total number of samples taken in the presented phase
OD_600_: Optical density at 600 nm
${\text{P}}_{{\text{g H}}_{2}}$: Partial pressure of hydrogen
PU-F68: Pluronic® F-68
PEM: Polymer electrolyte membrane
PtG: Power-to-gas
${\text{Y}}_{\text{rel H}_{2}}$: Relative conversion yield of hydrogen to methane
SD: Standard deviation
TRL: Technology readiness levels
TR: Tubular reactor
TFBR: Tubular foam-bed reactor
${\text{V}}_{{\text{CH}}_{4}}\text{/}{\text{V}}_{\text{R}}\text{/d}$: Volume of the methane per volume of the reactor per day
w/v: Weight per volume
