# Supplementary material for: Improved biological methanation using tubular foam-bed reactor
Source: Biotechnol Biofuels Bioprod. 2024 May 15;17:66. doi: 10.1186/s13068-024-02509-1 (PMC11097517; doi:10.1186/s13068-024-02509-1)
Supplement: Supplementary file 1 — Additional file1: S.1. Inoculation and biomass enrichment supplementary information includes preparation of the inoculum start-up and operation information of the biological methanation tubular reactor during different phases. Table S.1 Composition of the applied 1 × nutrient solution was as follows. To prepare the nutrient solution, a sterilized filtration unit was used. After preparing the nutrient mixture, the solution was either degassed with N2 for 5 min or kept overnight in the anaerobic workbench for the media of the tubular foam-bed bioreactor, which included 1.5 % (w/v) Pluronic® F-68 agent. In the final step and before usage, the sterilized Na2S·9H2O solution was added. In phase (I), the media included 10 % (v/v) formic acid as a co-carbon source. [file 13068_2024_2509_MOESM1_ESM.docx]

## **S.1** **Inoculation and biomass enrichment**

The mesophilic tubular reactor (TR) was inoculated with a mixture of biomass from a running laboratory-scale biological methanation reactor and an anaerobic digestion reactor (Temperature (T): 40°C). After inoculation, the starting OD_600_ was 0.7. To facilitate enrichment of hydrogenotrophic methanogens, formic acid (10% v/v) was added to the media as a co-carbon source during this phase. To achieve higher cell density in a shorter time, the harvest was collected in an anaerobic bottle and transferred to an anaerobic workbench (BugBox Baker Ruskinn Technology LTD, Bridgend, UK) as soon as it reached 50 mL. It was then transferred to a pre-anaerobe 50 mL Falcon tube, tightly closed, and centrifuged at (20 min,10,000×g). The supernatant was removed within the anaerobic workbench, and the precipitate was dissolved in 5 mL of supernatant and returned to the TR. The final OD_600_ in phase (I) reached approximately 2.7 at the end of this phase.

With the start of phase (II), 1.5% (w/v) of Pluronic® F-68 was added to the liquid phase of the reactor under mesophilic conditions at 40°C, and the reactor was changed to a tubular foam-bed bioreactor (TFBR). By applying biomass recycling in this phase, the OD_600_ finally reached 19.6. The highest biomass concentration recorded in this phase was during the partial H_2_ feeding experiment (OD_600_ = 26.8, dry weight = 12.9 g/L). The feeding media during phase (II) was approximately 6 mL/day (2× media, Tab. S.1).

In the starting phase (III), the TFBR temperature condition was changed from mesophilic (40°C) to thermophilic (55°C) at a rate of 5°C per week. When the temperature reached 55°C in phase (III), the biomass dry weight almost halved compared to the starting point. To compensate for the loss of necessary biomass, the TFBR was again inoculated with 20 ml of the thermophilic laboratory-scale anaerobic digester. The TFBR at 55°C started with an OD_600_ of 9.83 and ended at an OD_600_ of 23.92 after two months of operation. During the thermophilic conditions, to minimise water ingress into the system through media feeding, the 2× media was replaced with 4× media, and the feeding rate was eventually increased to 12 mL/day.

**Table S.1:** The composition of the applied 1× nutrient solution was as follows. To prepare the nutrient solution, a sterilised filtration unit was used. After preparing the nutrient mixture, the solution was either degassed with N_2_ for 5 minutes or kept overnight in the anaerobic workbench for the media of the tubular foam-bed bioreactor, which included 1.5% (w/v) Pluronic® F-68 agent. In the final step and before usage, the sterilised Na_2_S·9H_2_O solution was added. In phase (I), the media included 10% (v/v) formic acid as a co-carbon source.

| **Nutrient** | **Molecular Formula** | **Final Concentration (g/L)** |
| --- | --- | --- |
| Urea | CH_4_N_2_O | 2.56E+00 |
| Cysteine hydrochloride monohydrate | C_3_H_7_NO_2_S ∙ HCl ∙ H_2_O | 5.00E-01 |
| Calcium chloride dihydrate | CaCl_2_ ∙ 2H_2_O | 5.30E-02 |
| Dipotassium hydrogen phosphate | K_2_HPO_4_ | 2.20E+00 |
| Magnesium chloride hexahydrate | MgCl_2_ ∙ 6H_2_O | 5.06E-01 |
| Iron (II) sulphate heptahydrate | FeSO_4_ ∙ 7H_2_O | 5.12E-02 |
| Sodium chloride | NaCl | 7.00E-03 |
| Sodium sulphide | Na_2_S. 9H_2_O | 4.50E-01 |
| Cobalt chloride hexahydrate | CoCl_2_ ∙ 6H_2_O | 1.00E-02 |
| Copper (II) chloride dihydrate | CuCl_2_ ∙ 2H_2_O | 5.00E-04 |
| Boric acid | H_3_BO_3_ | 1.50E-02 |
| Manganese (II) chloride thetrahydratetetrahydrate | MnCl_2_ ∙ 4H_2_O | 1.50E-03 |
| Sodium molybdate dihydrate | Na_2_MoO_4_ ∙ 2H_2_O | 1.50E-03 |
| Sodium selenite | Na_2_SeO_3_ | 1.00E-03 |
| Nickel (II) chloride hexahydrate | NiCl_2_ ∙ 6H_2_O | 1.00E-03 |
| Zinc sulphate heptahydrate | ZnSO_4_ ∙ 7H_2_O | 3.60E-01 |
| Lipoic acid | C_8_H_14_O_2_S_2_ | 5.00E-06 |
| Pyridoxine hydrochloride | C_8_H_11_NO_3_ ∙ HCl | 1.00E-05 |
| Thiamine hydrochloride dihydrate | C_12_H_17_ClN_4_OS ∙ HCl ∙ 2H_2_O | 5.00E-06 |
| Riboflavin | C_17_H_20_N_4_O_6_ | 5.00E-06 |
| Vitamin B12 | C₆₃H₈₈CoN₁₄O₁₄P | 1.00E-07 |
| Nicotinic acid | C_6_H_5_NO_2_ | 5.00E-06 |
| D-Calcium-pantothenate | C_18_H_32_CaN_2_O_10_ | 5.00E-06 |
| Biotin | C_10_H_16_N_2_O_3_S | 2.00E-06 |
| Folic acid | C_19_H_19_N_7_O_6_ | 2.00E-06 |
| p-Aminobenzoic acid | C_7_H_7_NO_2_ | 5.00E-06 |
