# Supplementary material for: Improved biological methanation using tubular foam-bed reactor
Source: Biotechnol Biofuels Bioprod. 2024 May 15;17:66. doi: 10.1186/s13068-024-02509-1 (PMC11097517; doi:10.1186/s13068-024-02509-1)
Supplement: Supplementary file 2 — Additional file 2: S.2. Process parameter estimations include formulas for the calculation of methane production rate and substrate conversion efficiency for biological methanation. It also includes formulas to calculate H2 production based on Faraday’s law. [file 13068_2024_2509_MOESM2_ESM.docx]

## **S.2.Process parameter estimations**

The volume fraction of the different gases *i* in the input and the output ${(y}_{i})$ were calculated as

$\text{y}_{\text{i}}\text{=}\frac{\text{V}_{\text{i out}}}{\text{V}_{\text{T out}}}\text{ }\left( \frac{\text{L}_{\text{i}}}{\text{L}_{\text{T}}} \right)\text{×100 }\left( \text{\%} \right)\text{ }$ Eq. (S.1)

$V_{i out}$ (L): volume of component i in the outlet gas

$V_{T out}$ (L): total of volume of outlet gas in the normal condition.

The *MPR* provides information on the productivity of the reactor, which is normalised based on the reactive volume phase (tube volume) (V_R_) as follows based on Thema et al., 2019 defenitions:

$$\text{MPR}\text{ =}\frac{\text{V}_{\text{CH}\text{4 }\text{out}}\text{-}\text{V}_{\text{CH}\text{4}\text{ in}}\text{ }}{\left( \text{V}_{\text{R}}\text{×t} \right)}\text{ }\left( \frac{\text{L }\text{CH}_{\text{4}}}{\text{ }\text{L}_{\text{R}}\text{×d}} \right)\text{ }$$

Eq. (S.2)

$V_{{CH}_{4} out}$ (L): total volume of CH_4_ in the outlet

$V_{{CH}_{4} in}$ (L): total volume of CH_4_ in the inlet

V_R_ (L): tube volume

t (d): time.

The absolute CO_2_ conversion yield, $Y_{abs}{CO}_{2}$defines the CO_2_ conversion yield to CH_4_ and was calculated as represented by Thema et al., 2019

$$\text{Y}_{\text{abs}}\text{CO}_{\text{2}}\text{ =}\frac{\text{V}_{\text{CH}\text{4}\text{ Out}}\text{-}\text{V}_{\text{CH}\text{4}\text{ in}}}{\text{V}_{\text{CO}\text{2 }\text{in}}\text{ }}\text{ }\left( \frac{\text{L }\text{CH}_{\text{4}}}{\text{L }\text{CO}_{\text{2}}} \right)$$

Eq. (S.3)

$\text{V}_{\text{CO}\text{2 }\text{in}}$ (L): volume of input CO_2_ in the process.

The absolute conversion yield of H_2_ ($\text{Y}_{\text{abs}}\text{H}_{\text{2}}$) presented in Eq. (S.4) provides information about how much power is needed to produce a certain amount of CH_4_. The relative conversion yield $\text{Y}_{\text{rel}}\text{H}_{\text{2}}$ (Eq. S.5) defines the H_2_ conversion yield to CH_4_ and is obtained by division of the $\text{Y}_{\text{abs}}\text{H}_{\text{2}}$by 0.25 for a range 0 to 100% [1].

Eq. (S.4)

$$\text{Y}_{\text{abs}}\text{H}_{\text{2}}\text{ =}\frac{\text{V}_{\text{CH}\text{4}\text{ Out}}\text{-}\text{V}_{\text{CH}\text{4}\text{ in}}}{\text{V}_{\text{H}\text{2 }\text{in}}}\text{ }\left( \frac{\text{L }\text{CH}_{\text{4}}}{\text{L }\text{H}_{\text{2}}} \right)\text{ }$$

$\text{Y}_{\text{rel}}\text{H}_{\text{2}}\text{=}\frac{\text{Y}_{\text{abs}}\text{H}_{\text{2}}}{\text{0.25}}\text{ }\left( \frac{\text{L }\text{CH}_{\text{4}}}{\text{L }\text{H}_{\text{2}}} \right)\text{ }$ Eq. (S.5)

$\text{V}_{\text{H}_{\text{2}}\text{in}}$ (L): volume of input H_2_ in the process.

The H_2_ production by water PEM electrolyser calculated based on Faraday’s law and is presented as follows. Assuming the ideal gas law, hydrogen production of the polymer electrolyte membrane water electrolyser was calculated based on the second Faraday law as follows:

$\text{Q=}\text{I×t} \left( \text{A×s} \right)\text{ }\text{ }$ Eq. (S.6)

$\text{Q=}\text{n×z×F}\text{ }\left( \text{A×s} \right)$ Eq. (S.7)

*Q:* the electric charge ((A$\times$s) or C)

*I:* current (A)

*T:* time (s)

*n:* amount of substance (in this case H_2_ (mole)),

*z:* number of electrons required for separating a molecule of substance (z(H_2_) =2)

*F:* Faraday constant (96485 (A$\times$s)/mol).

The amount of H_2_ produced in 1 (s) and by applying 1 (A) ($\text{φ}_{\text{mole}}$) is calculated as follows:

$\text{φ}_{\text{mole}}\text{=}\frac{\text{n}}{\text{I×t}}\text{=}\frac{\text{1}}{\text{z×F}}\text{=}\frac{\text{1}}{\text{2×96485 }\frac{\text{(A×S)}}{\text{ mol}}}\text{=5.1812×}\text{10}^{\text{-6}}\text{ (}\text{mol}/{\left( \text{A×s} \right)\text{)}}$ Eq. (S.8)

Considering the molar volume of gas at standard conditions is 22.414 (L),$\varphi_{\mathrm{vol}}$ or the volume of H_2_ produced in 1 (min) by applying 1 (A) is calculated as follows:

$\text{φ}_{\text{vol}}\text{=}\text{ φ}_{\text{mole}}\text{×22.414}\left( \frac{\text{NL}}{\text{mole}} \right)\text{×60 (}\frac{\text{s}}{\text{min}}\text{) = 6.696×}\text{10}^{\text{-3}}\left( \text{NL}/\left( \text{A×} \text{min} \right) \right)\text{ }$ Eq. (S.9)

**References:**

[1] Thema, M., Weidlich, T., Hörl, M., Bellack, A., et al., Biological CO2-methanation: An approach to standardisation. *Energies* 2019, *12*.
