# Supplementary material for: Improved biological methanation using tubular foam-bed reactor
Source: Biotechnol Biofuels Bioprod. 2024 May 15;17:66. doi: 10.1186/s13068-024-02509-1 (PMC11097517; doi:10.1186/s13068-024-02509-1)
Supplement: Supplementary file 3 — Additional file 3: S.3. Process conversion yield during partial H2 feeding experiment includes Fig. S.1 Relative H2 conversion yield (${\text{Y}}_{\text{rel}}{{\text{H}}}_{2}$YrelH2) and absolute CO2 (${\text{Y}}_{\text{abs}}{{\text{CO}}}_{2}\text{)} \,$YabsCO2) conversion yield of biological methanation process in response to partial H2 feeding experiment in phase (II) within mesophilic tubular foam-bed bioreactor. [file 13068_2024_2509_MOESM3_ESM.docx]

## **S.3. Process conversion yield during partial H_2_ feeding experiment**


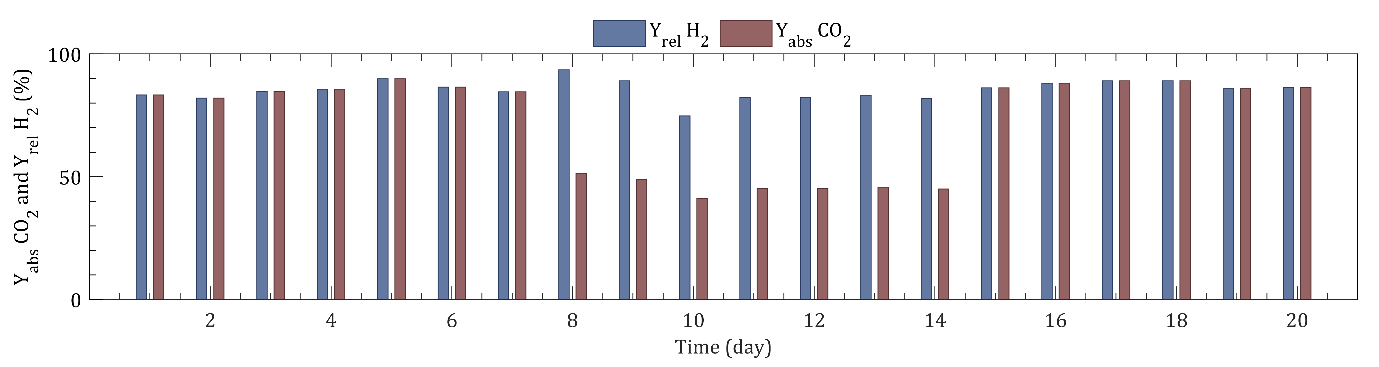
**Fig.S.1** Relative H_2_ conversion yield ($\text{Y}_{\text{rel}}\text{H}_{\text{2}}$) and absolute CO_2_ ($\text{Y}_{\text{abs}}\text{CO}_{\text{2}}\text{)}$conversion yield of biological methanation process in response to partial H_2_ feeding experiment in phase (II) within mesophilic tubular foam-bed bioreactor.
