# Supplementary material for: Improved biological methanation using tubular foam-bed reactor
Source: Biotechnol Biofuels Bioprod. 2024 May 15;17:66. doi: 10.1186/s13068-024-02509-1 (PMC11097517; doi:10.1186/s13068-024-02509-1)
Supplement: Supplementary file 4 — Additional file 4: S.4. Process results includes Fig. S.2 Time course of process results over a year considering different phases of the bioreactor. The Box–Whisker plots are produced using the average value of each day for every week. Different phases are separated using dash lines, and partial H2 feeding experiments are indicated by dash–dot lines during phase (II). In phase (I), a tubular reactor in mesophilic conditions was operated, whereas a tubular foam-bed reactor was operated in mesophilic and thermophilic conditions in phases II and III, respectively. (a) Daily methane production rate (MPR), (b) Volume fraction of CH4 in the outlet gas, (c) Loading of CO2 (L/d), (d) Loading of H2 (L/d). [file 13068_2024_2509_MOESM4_ESM.docx]

##
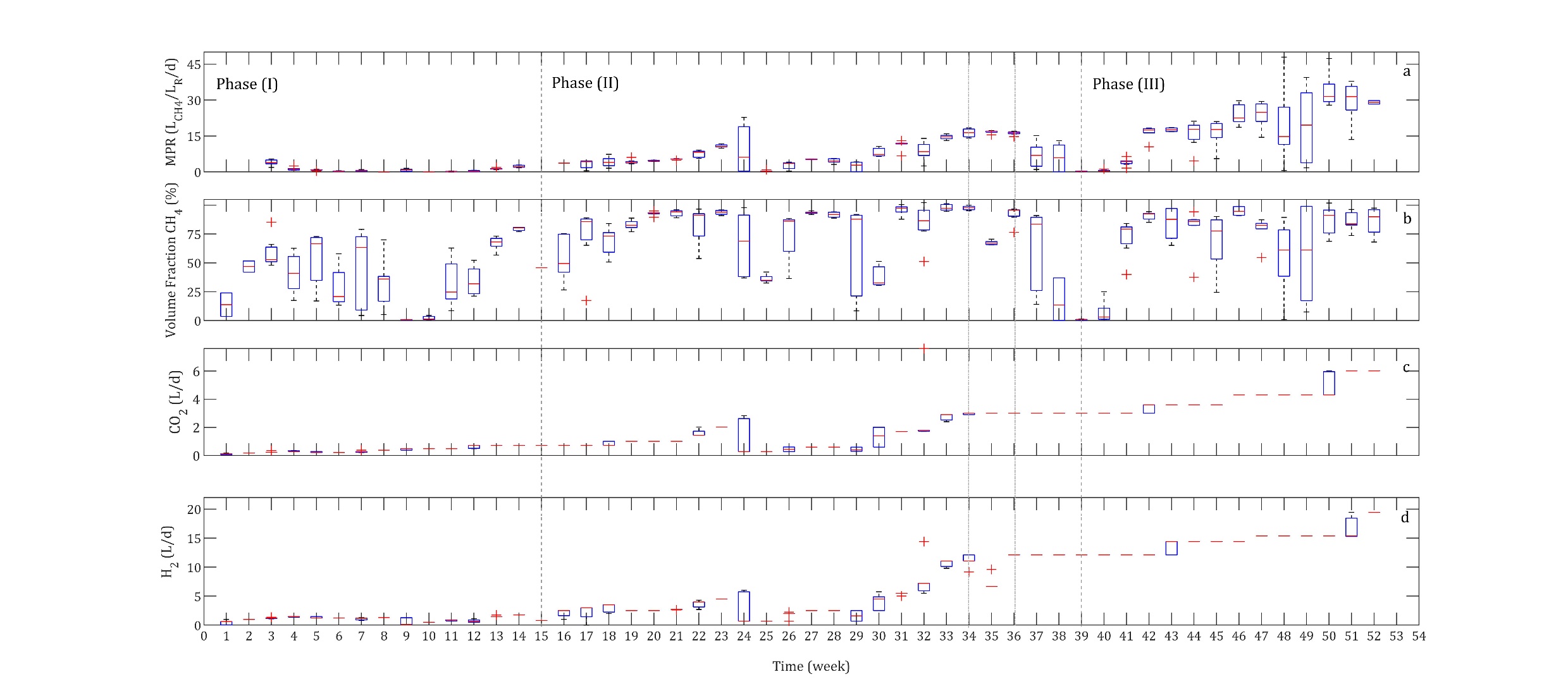
**S.4. Process result**

**Fig.S.2** Time course of process results over a year considering different phases of the bioreactor. The box-whisker plots are produced using the average value of each day for every week. Different phases are separated using dash lines, and partial H_2_ feeding experiments are indicated by dash-dot lines during phase (II). In phase (I), a tubular reactor in mesophilic conditions was operated, whereas a tubular foam-bed reactor was operated in mesophilic and thermophilic conditions in phases II and III, respectively. (a) Daily methane production rate (*MPR*), (b) Volume fraction of CH_4_ in the outlet gas, (c) Loading of CO_2_ (L/d), (d) Loading of H_2_ (L/d).
